# Identification and Distribution of Novel Metabolites of Lolitrem B in Mice by High-Resolution Mass Spectrometry

**DOI:** 10.3390/molecules25020372

**Published:** 2020-01-16

**Authors:** Priyanka Reddy, Aaron Elkins, Joanne Hemsworth, Kathryn Guthridge, Simone Vassiliadis, Elizabeth Read, German Spangenberg, Simone Rochfort

**Affiliations:** 1 Agriculture Victoria, AgriBio, Centre for AgriBioscience, Bundoora, VIC 3083, Australia; aaron.elkins@agriculture.vic.gov.au (A.E.); joanne.hemsworth@agriculture.vic.gov.au (J.H.); kathryn.guthridge@agriculture.vic.gov.au (K.G.); simone.vassiliadis@agriculture.vic.gov.au (S.V.); l.read@latrobe.edu.au (E.R.); german.spangenberg@agriculture.vic.gov.au (G.S.); simone.rochfort@agriculture.vic.gov.au (S.R.); 2 School of Applied Systems Biology, La Trobe University, Bundoora, VIC 3083, Australia

**Keywords:** lolitrem B, metabolism, mycotoxin, endophyte

## Abstract

Lolitrem B is the most potent indole-diterpene mycotoxin produced by *Epichloë festucae* var. *lolii* (termed *Lp*TG-1), with severe intoxication cases reported in livestock. To date, there are no in vivo metabolism studies conducted for the mycotoxin. A mouse model assay established for assessing toxicity of indole-diterpenes was used to investigate metabolic products of lolitrem B. Mice were administered lolitrem B at 0.5 and 2.0 mg/kg body weight (b.wt) intraperitoneally before body and brain tissues were collected at 6 h and 24 h post-treatment. Samples were cryoground and subjected to a biphasic or monophasic extraction. The aqueous and lipophilic phases were analysed using liquid chromatography high-resolution mass spectrometry (LC–HRMS); data analysis was performed with Compound Discoverer™ software. A total of 10 novel phase I metabolic products were identified in the lipophilic phase and their distribution in the liver, kidney and various brain regions are described. The biotransformation products of lolitrem B were found to be present in low levels in the brain. Based on structure–activity postulations, six of these may contribute towards the protracted tremors exhibited by lolitrem B-exposed animals.

## 1. Introduction

**Figure 1:**
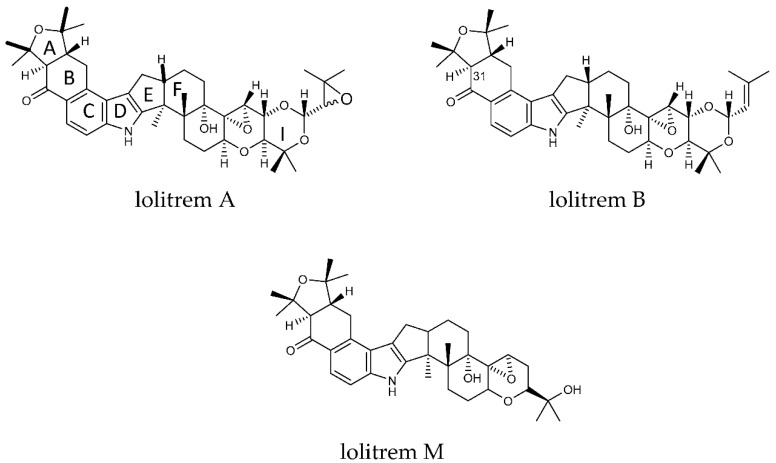
Structures of lolitrem A, B and M.

Many mycotoxins are contaminants of agricultural products found in animal feed and human food [1,2,3,4,5,6]. The adverse effects of low-level chronic exposure of mycotoxins in foodstuffs is well documented [2,3,4,5,6]. The mycotoxin lolitrem B (Figure 1) is the major compound responsible for ryegrass staggers and the most abundant indole-diterpene produced by perennial ryegrass endophyte *Epichloë festucae* var. *lolii* (also termed *Lp*TG-1). Perennial ryegrass is a temperate pasture grass that is commonly used for forage in countries including Northern Europe, Pacific North West of USA, Japan, South-eastern Australia, and New Zealand [7,8]. The neurological signs associated with perennial ryegrass toxicosis (PRGT) has been reported in Australia, New Zealand, Pacific northwest of USA and Europe [7]. The clinical signs of PRGT include head tremor and incoordination that is worsened with movement or exercise [9]. Currently, outside of farm grazing management, the only safety measure to prevent PRGT applies to conserved fodders, by quantitative determination of lolitrem B concentrations in straw destined for the export market. For example, Japan set threshold levels of lolitrem B at 1800–2000 parts per billion (ppb) as the maximum allowable limit in imported feed (straw) [10,11]. This threshold was established from field-based behavioural observations, where levels above 1800 ppb were implicated in naturally occurring outbreaks of perennial ryegrass toxicosis (PRGT).

The lipophilic nature of lolitrem B results in the accumulation of the toxin in fatty tissue. This was found to occur in sheep grazing on herbage containing 3400 ppb of lolitrem B [12] and was also present at low levels in subcutaneous and perirenal fat of cattle fed with straw containing 1200 ppb of lolitrem B; however, no clinical symptoms were exhibited [11]. The bioaccumulation of lolitrem B is also known to occur in brain tissue of animals exposed to low levels (0.5 mg/kg b.wt) of lolitrem B, as demonstrated in the small animal model assay used in this study [13]. In 2013, Finch et al. showed that cattle grazing old naturalized perennial ryegrass pastures containing the Standard Endophyte (SE) strain, secreted 0.23% of the lolitrem B ingested, into milk [14]. The concentration of lolitrem B found in the fat or milk was not high enough to elicit an observable behavioural response in the animal [14]. However, the current behavioural measures used to assess PRGT, may not be sufficient or sensitive enough to detect subtle metabolic perturbations that may result from extended exposure to the fungal mycotoxin [13]. Thus, further investigations into the long-term sub-clinical effects of lolitrem B in humans and pasture-fed livestock are warranted.

Concentrations of other members belonging to the lolitrem B family of indole diterpenoid toxins have also not been investigated in meat or milk to date. These structurally similar intermediates in the indole-diterpene pathway are present in endophyte-infected perennial ryegrass and tall fescue marketed pasture varieties. Although most marketed endophytes produce very low or no lolitrem B, some do produce intermediates in the pathway. Additionally, a blockade of the production of downstream components like lolitrem B can result in the overproduction of its upstream counterparts [15,16]. It is therefore important that these intermediate pathway members also be thoroughly investigated for toxicity, particularly those that are identified as tremorgenic [7].

Many tremorgenic indole-diterpenes such as janthitrems, aflatrem, paspalinine, paxilline, and verruculogen are lipophilic molecules. Research suggests that these compounds cross the blood-brain barrier and thereby gain access to the brain, disrupting the central nervous system with similar effect to lolitrem B [17]. The presence of these related indole-diterpenes in marketed forage grasses, such as AR37, could therefore be problematic. Although epoxy-janthitrems are the likely cause of ryegrass staggers in livestock grazing AR37 endophyte-infected pasture, it is not as potent as lolitrem B [12,18,19]. The lack of scientific data on these compounds has led to public health concerns focusing on the safety of lolitrem B and the allowable thresholds currently regarded as safe for humans, with little focus on metabolic precursors [11]. Thus, an investigation on the metabolism of the indole-diterpenes is warranted since they continue to be present in human food.

The tremorgenic indole-diterpenes are suggested to act via antagonism of large conductance potassium channels (BK channels) [7,20]. These channels are found ubiquitously throughout the brain but are highly expressed in both the cerebellum and cerebral hemispheres [21,22] and are suggested to constitute a primary target. More recently, mice exposed to lolitrem B have shown bioaccumulation and metabolic disruption in the brain, particularly a dysregulation in the catecholaminergic pathway [13].

The effect of tremorgenic indole-diterpenes on behaviour has been shown to be reversible; animals show recovery when they are no longer exposed to the toxin, suggesting that metabolic breakdown or receptor recycling is occurring to remove these strongly bound toxins from the synapse [23,24,25]. However, the mechanism(s) of neuronal detoxification have not yet been elucidated.

Despite substantial evidence pertaining to lolitrem B and its antagonistic effects on BK channels in the brain, the identification and distribution of the bioactive metabolic products have not been studied. Therefore, to identify the metabolic products of lolitrem B and its distribution in the body and brain, predicted biotransformation products were first investigated in liver extracts from the in vivo mouse model assay. Based on the transformation products identified, an inclusion list was generated, and data were acquired using Liquid Chromatography High-Resolution Mass Spectrometry (LC–HRMS) in the full-scan MS/data-dependent MS^2^ (ddMS^2^) mode. Here, we provide identification of ten phase I metabolites and their proposed structural configuration. The identification and distribution of the lolitrem B metabolic products in the liver, kidney and various brain regions were then determined and found to be present in these tissues. Six of these compounds are thought to be tremorgenic based on hypotheses related to structure–activity postulations [26] and are very likely to be present in animal products for human consumption. Thus, it is important to establish the risks associated with exposure of these compounds to humans as well as grazing livestock.

## 2. Results and Discussion

**Figure 2:**
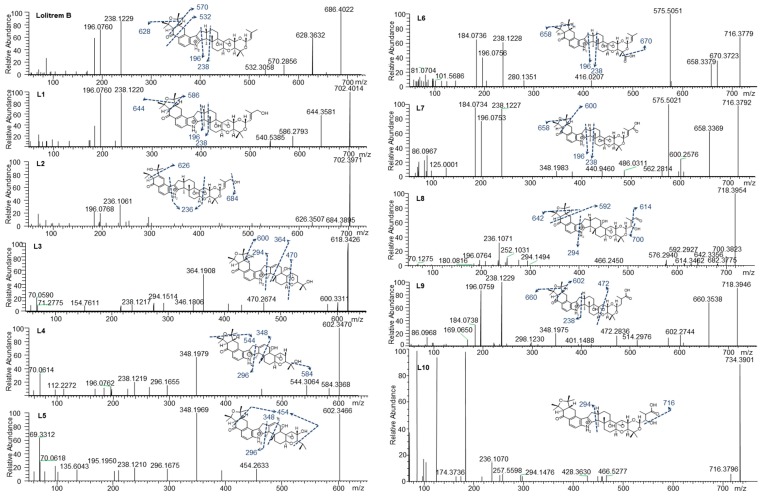
Lolitrem B, L1 to L10 MS^2^ spectra and proposed structures and fragmentation pattern of the respective metabolites.

To determine the MS/MS fragmentation pattern of lolitrem B, the purified compound was analysed by LC–MS/MS. Lolitrem B eluted at 8.56 min ([M + H]^+^, *m*/*z* 686.4022) and the mass spectrum indicated fragmentation of the tetramethyltetrahydrofuran ring and loss of a C_3_H_6_O moiety to give the highest mass fragment (*m*/*z* 628.3632). Dehydrogenation and opening of the adjacent cyclohexone ring results in the loss of C_7_H_16_O giving rise to the fragment *m*/*z* 570.2856. Further fragmentation of the cyclohexone ring with loss of C_9_H_14_O_2_ (*m*/*z* 532.3058) was also identified. The most intense fragments produced include *m*/*z* 238.1229 and 196.0760 (Figure 2) which are common in many of the metabolic products that have been identified in this study.

Phase I and phase II metabolites were targeted (Supplementary Table S2) in liver tissue extracts from lolitrem B treated male mice, to identify metabolic products of the tremorgenic mycotoxin. Although both phase I and phase II metabolites were detected, the abundances for the phase I metabolites were sufficient for data-dependent acquisition. The ten metabolic products, L1 to L10, identified are phase I reactions of lolitrem B (Table 1) resulting from hydroxylations, oxidations and dehydrogenation reactions occurring at the isoprene chain and the tetrahydrotetrafuran ring as the dominant site of metabolism (Figure 2).

**Figure 3:**
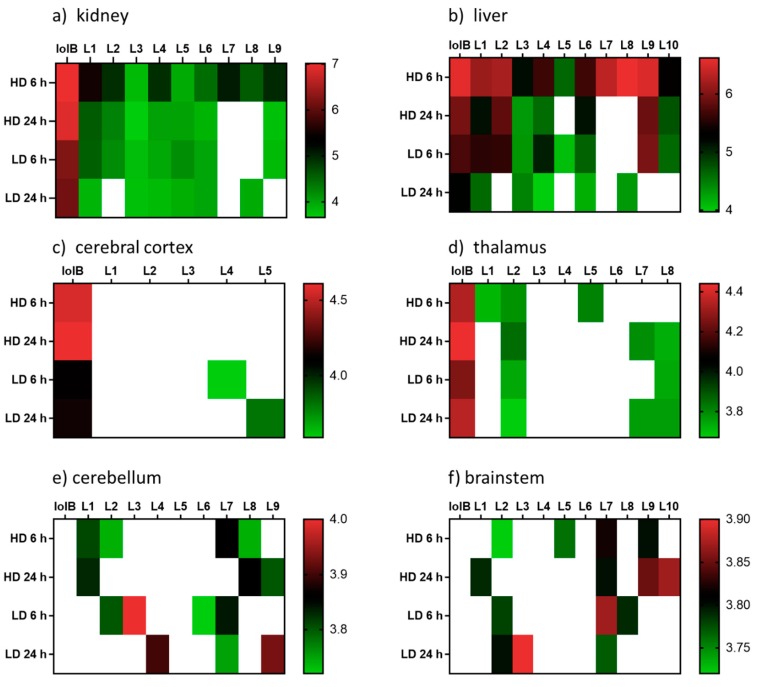
Heatmap displaying the relative abundances (log10 transformed) of lolitrem B biotransformation products distributed in the body: (**a**) kidney (**b**) liver and brain tissue: (**c**) cerebral cortex (**d**) thalamus (**e**) cerebellum (**f**) brainstem of mice exposed to lolitrem B at high dose (HD) (2.0 mg/kg b.wt) and low dose (LD) (0.5 mg/kg b.wt) at time points 6 h and 24 h post treatment.

The key attributes postulated for the tremorgen lolitrem B as a potent neurotoxin include the presence of the acetal-linked isoprene unit (Ring I) of the molecule, and the presence of the A/B rings (Figure 1) and the position of the hydrogens in the junction of the A/B rings [25,26]. The presence of the A/B rings are thought to be responsible for the slow-onset and prolonged tremorgenic activity of the indole-diterpene toxins [25,26]. According to these key postulations regarding the structure–activity relationship, the biotransformation products L1, L6, L7, L8, L9 and L10 (Figure 2) are likely to be tremorgenic. The addition of hydroxyl and/or carbonyl groups to the isoprene unit of these metabolic products may not affect tremorgenic potency. For example, lolitrem A differs from lolitrem B, in the epoxidation of the isoprene unit; however, the tremorgenic potency remains the same. Although the lolitrem B biotransformation products were not present in many of the replicates (Supplementary Table S3) and were low in abundance in the brain (Figure 3), these phase I metabolites may contribute towards to the prolonged tremorgenicity observed in animals. 

While addition to the isoprene unit may not affect tremorgenicity the loss of the isoprene unit and opening of the attached ring may inhibit the tremorgenic potency, as seen in lolitrem M. Similarly, lolitrem B loses its tremorgenicity when it degrades to lolitriol [25,27]. Thus, L2, L3, L4 and L5 may not be tremorgenic metabolic products.

L1 (*m*/*z* 702.4014) and L2 (*m*/*z* 702.3971) were the result of lolitrem B hydroxylation (+O) as indicated by the +15.9950 Da mass shift from the parent ion. The addition of the hydroxyl moieties resulted in earlier elution times for L1 and L2 (7.34 and 7.83 min) compared to lolitrem B (8.56 min). The mass difference observed in L1 fragments *m*/*z* 644.3581 and *m*/z 586.2793 were consistent with hydroxylation of lolitrem B. The most intense signals produced by L1, *m*/*z* 238.1220 and *m*/*z* 196.0760, were similar to L2 and consistent with lolitrem B fragment ions. The fragmentation pattern of L1 suggests hydroxylation of the terminal isoprene. The L2 fragment *m*/*z* 684.3895 suggests neutral loss of H_2_O while *m*/*z* 626.3507 and *m*/*z* 236.1061 of L2 suggests the opening of the furan ring and the dehydrogenation of the fused cyclohexone ring. The proposed biotransformation product for L1 and L2 are shown in Figure 2.

L3 (*m*/*z* 618.3426) elutes at 6.46 min and the MS^2^ spectrum indicates a neutral loss of H_2_O, *m*/*z* 600.3311, and a subsequent loss of the tetrahydropyran ring, *m*/*z* 470.2674. The fragmentation pattern (in particular the ions representing fragmentation at either end of the molecule, *m/z* 470.2674 and *m*/*z* 294.1514) suggest that the terminal I ring has been degraded in this metabolite, removing the ether linked isoprene subunit and leaving the residual isopropyl moiety attached to ring H (Figure 1). Together, the fragments *m*/*z* 346.1806 and *m*/*z* 294.1514 indicate the presence of the double bond (in ring F) (Figure 1) adjacent to the central 5-membered ring, creating a new site around which fragmentation occurs. The intense ion *m*/*z* 364.1908 suggests that hydroxylation most likely occurs on the adjoining cyclohexone and furan ring. The diagnostic ion *m*/*z* 238.1217 further supports the proposed structure of L3 (Figure 2).

L4 (*m*/*z* 602.3470) and L5 (*m*/*z* 602.3466) elute at 7.38 and 7.76 min respectively. Like L3, these metabolites have lost Ring I, yet they are differentiated by where the hydroxy moiety is retained. L4, like L3, retains the hydroxy such that there is a terminal isopropanol group (*m*/*z* 584.3368), whereas L5 retains the oxygen directly attached to Ring H (*m*/*z* 454.2633). The fragmentation of the cyclohexone ring, loss of the terminal dimethyl group and subsequent hydrogenation steps result in the *m*/*z* 454.2633 ion. The position of the double bond is consistent with L3 as suggested by the intense fragment ion produced at *m*/*z* 348.1969 as well as *m*/*z* 296.1675, comparable to what is seen in the MS^2^ spectrum for L4. The *m*/*z* 544.3064 in L4 showed the same mass difference (-C_3_H_6_O) from the parent ion, consistent with lolitrem B fragmentation, and is further supported by the presence of the diagnostic ion *m*/*z* 238.1219 and *m*/*z* 196.0762. 

L6 (*m*/*z* 716.3779) and L7 (*m*/*z* 716.3792) are likely regioisomers as they possess the same predicted molecular formula as shown in Table 1; however, they elute at 7.66 and 7.77 min respectively. Aliphatic carboxylation of a terminal methyl group on Ring I is likely for L6 and L7. The fragmentation spectrum displayed the characteristic loss of *m*/*z* 58 (*m*/*z* 658.3379) as well as the diagnostic ions *m*/*z* 238.1228 and *m*/*z* 196.0756 common to lolitrem B. The MS^2^ spectrum indicated carboxylic acid loss and dehydrogenation in L6 resulting in *m*/*z* 670.3723, that further confirmed the carboxylation. The fragmentation spectrum of the aliphatic carboxylation isomer L7, displayed the consistent loss of *m*/*z* 58 (*m*/*z* 658.3369) and *m*/*z* 116 (600.2576) as well as the diagnostic ions *m*/*z* 238.1227 and *m*/*z* 196.0753. 

L8 (*m*/*z* 718.3954) and L9 (*m*/*z* 718.3946) possess the same predicted molecular formula. The retention times and mass spectra indicate structural variation. L8 elutes at 6.45 min and all fragments produced are subsequent to the neutral loss of water (-H_2_O) *m/z* 700.3823. The *m/z* 642.3356 ion indicates the characteristic loss of (-C_3_H_6_O) and the relatively low intense ion *m/z* 614.3462 represents the subsequent loss of the carbonyl group. Also, the ion *m/z* 294.1494 characterises fragmentation adjacent to the pyrrole ring and indicates carboxylation on the terpene chain. Complete loss of the tetramethyltetrahydrofuran ring results in *m*/*z* 592.2927. The spectrum also results in the diagnostic fragment *m*/*z* 196.0764 and the dehydrogenation modification resulting in *m/z* 236.1071. 

L9 elutes at 7.73 min and most likely represents carboxylation, as it retains the less labile hydroxy group. The characteristic losses (-C_3_H_6_O) *m*/*z* 660.3538 and (-C_7_H_16_O) *m*/*z* 602.2744 as well as fragments ions *m*/*z* 238.1229 and *m*/*z* 196.0759 diagnostic to lolitrem B were produced. Fragmentation and dehydrogenation resulted in *m*/*z* 348.1975 and *m*/*z* 472.2836 indicating hydroxylation and carbonylation on the terpene chain.

L10 (*m*/*z* 734.3901) elutes at 6.89 min and the molecular formula suggests trioxidation of lolitrem B. Fragment *m*/*z* 716.3796 indicates loss of a hydroxyl group. The presence of the diagnostic product ions *m*/*z* 236.1070 and 294.1476 further confirmed the structural origin of the biotransformation product. The proposed structure is consistent with available data; however, it is also possible that oxidation occurred in some of the more central rings resulting in the decreased fragmentation seen in the MS^2^ spectrum.

The distribution of the lolitrem B metabolites identified in this study were examined in the brain (cerebral cortex, thalamus, cerebellum and brainstem) and body tissue (kidney and liver) of lolitrem B treated mice (Figure 3). Metabolism of complex compounds occur primarily in the liver. The kidneys are responsible for the elimination of metabolic waste. The abundances of the metabolites L1 to L10 in liver 6 h at high dose were between 3.8-fold to 24.2-fold higher than liver 24 h at high dose. A similar trend was also observed in kidney 6 h, showing 1.4-fold to 16-fold higher levels than kidney 24 h in L1 to L4, L7 and L9 metabolites. The presence of lolitrem B metabolites L1 to L5, L6 and L9 in the kidney and liver at 6 h as well as 24 h indicate prolonged metabolism and excretion of the biotransformation products. With the exception of L10, all other lolitrem metabolites are transported systemically after metabolism of lolitrem B and have been identified in the brain tissue. Similar to lolitrem B, high levels of the metabolic products are present at 6 h (peak neurotoxic activity) compared to 24 h in the kidney and liver and there is a clear dose-dependent effect and clear profile of excretion over time for all its metabolic products [13].

Many of the lolitrem metabolites are either not detected (ND) in the brain or only identified in one, or at most four, replicates (Supplemetary Table S3). This may be related to a combination of low abundance and poor detection of the compound in the mass spectrometer. Despite the low incidence of the compound in the brain, the contribution of these conceivably active metabolites to the blockade of BK channels throughout the body and brain cannot be disregarded. 

In conclusion, this is the first study to report the metabolism of any indole-diterpene compound produced by the *Epichloë* endophyte using an in vivo mouse model assay. The relative abundances of the 10 phase I metabolites throughout the body and brain give an indication on the compounds that leave the liver, enter the systemic circulation and perturb metabolic processes in the central and peripheral nervous system. The proposed chemical structures of the novel metabolites in this study provide insight into the mechanism and metabolic site of transformation for the indole-diterpene alkaloids. 

## 3. Materials and Methods

The methodology used in this study is based on workflows adapted from the literature [28,29,30].

### 3.1. Ethical Approval

All mouse studies were approved by the La Trobe University Animal Ethics Committee (Protocol number 15–87) and were conducted in accordance with the Australian Code of Practice for the Care and Use of Animals for Scientific Purposes set out by the National Health and Medical Research Council of Australia [13]. The mice were housed in groups of two to four during the experimental period in individually-ventilated cages (Tecniplast, Buguggiate, Italy) with standard pellet food and water available ad libitum. Ambient temperature of housing and testing rooms was 21 ± 2 °C and mice were housed under a 12 h light–dark cycle (lights on at 07:00). A total of 184 male 8–9-week-old C57Bl/6J mice were sourced from a breeding colony at the Walter and Eliza Hall Institute of Medical Research, Melbourne, Victoria. Animals were allowed to acclimatise to the facility conditions for a period of 1 week prior to experiments [13].

### 3.2. Tissue Extracts

The mouse tissue samples collected in this study were derived from research conducted in Reddy et al. [13]. The body and brain tissue extracts used in this study were generated as per methods described in Reddy et al. [13]. Briefly, kidney, liver and brain regions (cerebral cortex, thalamus, brainstem and cerebellum) of mice exposed to 0.5 mg/kg b.wt or 2.0 mg/kg b.wt doses of lolitrem B were collected at either 6 h or 24 h post-treatment and compared to untreated controls or vehicle injected controls 9:1 (*v*/*v*) dimethylsulfoxide (DMSO): H_2_O, with *n* = 8 for each treatment and time point. The dosages 0.5 and 2.0 mg/kg correspond to hypothesized sub-clinical (low) doses and known disease-inducing (high) concentrations subsequent to ingestion in pasture-fed herbivores [13,31]. 

Animals were euthanized by cervical dislocation and brain and body tissues were collected. Samples were snap-frozen in liquid nitrogen (N_2_) before storage at −80 °C for analysis. Frozen tissue samples were cryoground, and the fine powder was stored at −80 °C. Kidney and liver samples (50–52 mg) as well as cerebral cortex and thalamus (20–22 mg) samples were each weighed in 2 mL Eppendorf tubes. Samples were extracted using a modified Bligh-Dyer extraction method [32]. Briefly, methanol: H_2_O (1.6:0.6, *v*/*v*) was added to tissue powder and vortex-mixed prior to addition of dichloromethane (DCM). Samples were then sonicated on ice before addition of 1:1, *v*/*v* DCM: H_2_O. The aqueous and lipophilic solvent layers were then transferred to separate tubes and evaporated under a stream of N_2_. The lipophilic extract was reconstituted in 200 µL and 50 µL aliquots were subsequently transferred to HPLC vials with inserts. In this study, the lipophilic liver extracts were further concentrated to increase the abundance of the targeted metabolites and the respective MS^2^ fragments of the biotransformation products. Thus, a 40 µL aliquot of the 200 µL concentrated extract was pooled for each treatment group and evaporated under a stream of N_2_ and reconstituted in 150 µL of 1:1 *v*/*v* DCM: methanol.

The cerebellum and brainstem were smaller (30–70 mg) than other tissues and thus were directly placed in cryotubes prefilled with ceramic beads. For homogenization, 1 mL of cold 4:1 *v*/*v* methanol: H_2_O was added to each sample and homogenized at −20 °C, with a tissue homogenizer fitted to a cooling system, at a speed of 5500 rpm, with three consecutive 20 sec cycles and 30 sec intervals. The cerebellum and brainstem tissues were subsequently extracted with 4:1 *v*/*v* methanol: H_2_O (40 µL/mg sample) and 50 µL aliquots of the aqueous phase was transferred into HPLC vials for LCMS analysis. 

### 3.3. Liquid Chromatography–High-Resolution Tandem Mass Spectrometry Parameters

Chromatographic separation and MS detection were performed on samples using methods described in Reddy et al. [13]. Briefly, a Vanquish Ultra-High-Performance Liquid Chromatography system (Thermo Fisher Scientific, Bremen) was coupled to a QExactive Plus mass spectrometer (LC–MS) (Thermo Fisher, Waltham, MA, USA; Thermo, Bremen, Germany). The MS data were acquired with polarity switching between the positive and negative mode over a mass range of 70–1200 amu with resolution set at 35,000. Separation was achieved using an Agilent 150 × 2.1 mm, 3.5 µm Zorbax Eclipse XDB-C8 column with a linear gradient of 2% to 100% B over 11 min with mobile phase, A (0.1% formic acid in H_2_O) and B (0.1% formic acid in 55% isopropanol (IPA) in acetonitrile) at a flow rate of 0.5 mL/min. Injection volume was 3 µL.

### 3.4. Metabolite Identification

Lolitrem B was isolated as per Reddy et al. [33] and used for identification purposes in the current study. Raw LC–MS data of the control and treatment cohorts of the aqueous and lipophilic sample extracts were imported into Compound Discoverer™ software (v. 3.0; Thermo Scientific, Fremont, CA, USA) to identify lolitrem B metabolites. The expected phase I and phase II transformations (as per parameters reported in Supplementary Table S1) were generated and identified from the MS spectra using the following settings: mass tolerance 5 ppm; intensity tolerance (isotopes), 30%; minimum peak intensity, 1000. All metabolic products of lolitrem B were identified in the lipophilic phase.

Data were acquired in the full-scan MS/data-dependent MS^2^ (ddMS^2^) mode of the concentrated lipid extract. MS cycles were composed of 1 FullMS and up to 5 ddMS^2^. The 5 ions with the most intense signal detected in the full MS survey scan (intensity threshold 4.0 × 10^4^) triggered a MS^2^ event at the peak apex with an isolation window of 0.4 *m*/*z*. A 2.0 s delay was required for the same ion to trigger a new MS^2^ event (dynamic exclusion), a short dynamic exclusion was selected so MS^2^ fragmentation data were generated for closely eluting isobaric compounds. Full MS scans were acquired from *m*/*z* 300 to 800 with a resolution of 70,000 (full width at half maximum, FWHM, at *m*/*z* 200); automatic gain control (AGC) target was 3 × 10^6^; maximum injection time (IT) 200 ms. Scans (ddMS^2^) were acquired at a resolution of 17,500, the AGC target was 1 × 10^5^, and the maximum IT was 50 ms. Ions were fragmented with stepped collision energy (20, 40 and 60%).

These data were processed in Compound discoverer™ (Thermo Scientific) to generate a target list of 64 potential metabolites (Supplementary Table S2). An inclusion list targeting the ions of interest were analysed by full-scan MS/ddMS^2^ on the concentrated liver extract to determine if they were metabolic products of lolitrem B. 

### 3.5. Metabolite Distribution

Kidney, liver and brain tissue samples were analysed using Thermo Xcalibur Qual Browser v.2.3.26 (Thermo Fisher Scientific™) to determine the distribution of the biotransformation products identified in Compound Discoverer. Assessment of peak retention time and ion extraction window (*m*/*z*) confirmed the presence of targeted lolitrem B metabolic products (Supplementary Table S3). The relative abundances of the metabolic products were normalised by log10 transformation and heatmaps were generated using GraphPad Prism 8.2.1 (GraphPad Software, Inc., La Jolla, CA, USA).

## Figures and Tables

**Table 1:** Accurate mass molecular ion, retention time, elemental composition, nominal mass for diagnostic product ions.

Metabolite	Biotransformation	[M + H]^+^ *m*/*z*	Retention Time, Min	Mass Error, ppm	Diagnostic Product Ions, *m*/*z*
Lolitrem B	C_42_H_56_NO_7_	686.4036	8.56	−4.239	628.3632 570.2856, 532.3058, 238.1229, 196.0760
L1	C_42_H_56_NO_8_	702.4014	7.34	1.888	644.3581, 586.2793, 540.5385, 238.1220, 196.0760
L2	C_42_H_56_NO_8_	702.3971	7.83	−0.718	684.3895, 626.3507, 236.1061, 196.0768
L3	C_37_H_48_NO_7_	618.3426	6.46	0.098	600.3311, 470.2674, 364.1908, 294.1514, 238.1217
L4	C_37_H_48_NO_6_	602.3470	7.38	−0.738	584.3368, 544.3064, 348.1979, 296.1655, 238.1219, 196.0762
L5	C_37_H_48_NO_6_	602.3466	7.72	0.275	454.2633, 348.1969, 296.1675, 238.1210, 195.1950
L6	C_42_H_54_NO_9_	716.3779	7.77	−1.817	670.3723, 658.3379, 238.1228, 196.0756
L7	C_42_H_54_NO_9_	716.3792	7.66	−0.221	658.3369, 600.2576, 238.1227, 196.0753
L8	C_42_H_56_NO_9_	718.3954	6.45	−0.346	700.3823, 642.3355, 592.2927, 236.1069, 196.0764
L9	C_42_H_56_NO_9_	718.3946	7.73	−0.513	660.3538, 602.2744, 472.2836, 348.1975, 238.1229, 196.0759
L10	C_42_H_56_NO_10_	734.3923	6.89	0.281	716.3796, 236.1070, 294.1476, 236.1070

## Supplementary Materials

The following are available online. Table S1: Parameters used to identify lolitrem B metabolites with Compound Discoverer™; Table S2: Data-dependent acquisition during analysis of liver samples with LC–MS on Thermo QExactive; Table S3. The mean relative abundance of lolitrem B biotransformation products distributed in the body and brain tissue of mice exposed to lolitrem B at high (2.0 mg/kg b.wt) and low (0.5 mg/kg b.wt) doses at time points 6 h and 24 h post treatment.
