# Supplementary material for: Identification and Distribution of Novel Metabolites of Lolitrem B in Mice by High-Resolution Mass Spectrometry
Source: Molecules. 2020 Jan 16;25(2):372. doi: 10.3390/molecules25020372 (PMC7024290; doi:10.3390/molecules25020372)
Supplement: Supplementary file 1 [file molecules-25-00372-s001.pdf]

Supplementary Materials: Identification and Distribution of Novel Metabolites of Lolitrem B in Mice by High-Resolution Mass Spectrometry

Priyanka Reddy [1,2,*], Aaron Elkins[1], Joanne Hemsworth[1], Kathryn Guthridge[1], Simone Vassiliadis[1], Elizabeth Read[1], German Spangenberg[1,2], Simone Rochfort[1,2]

Table S1. Parameters used to identify lolitrem B metabolites with Compound Discoverer™

	Lolitrem B ($C_{42}H_{55}NO_7$)
phase I transformations	**Oxidation, reduction, dehydration, dehydrogenation, hydration and reduction**
phase II transformations	**acetylation, glucuronide conjugation, methylation and sulfation**
maximum number of dealkylation steps	**1**
maximum number of phase II reactions	**1**
maximum number of reactions	**5**

Table S2. Data-dependent acquisition during analysis of liver samples with LCMS on Thermo QExactive.

Mass [m/z]	Polarity	Start [min]	End [min]	Mass [m/z]	Polarity	Start [min]	End [min]
**602.34692**	Positive	6.38	8.38	**716.41278**	Positive	7.77	9.77
**618.34125**	Positive	5.47	7.47	**718.39233**	Positive	5.75	7.75
**620.35864**	Positive	5.99	7.99	**718.39368**	Positive	6.22	8.22
**622.37103**	Positive	7.68	9.68	**718.39386**	Positive	6.76	8.76
**636.35034**	Positive	5.44	7.44	**718.39423**	Positive	5.47	7.47
**636.35406**	Positive	4.83	6.83	**718.42853**	Positive	7.79	9.89
**650.38513**	Positive	7.96	9.96	**718.42926**	Positive	7.68	9.68
**654.41608**	Positive	7.71	9.71	**718.42938**	Positive	7.85	9.85
**655.40021**	Positive	7.72	9.72	**722.42444**	Positive	7.88	9.88
**662.37012**	Positive	7.79	9.79	**726.39746**	Positive	7.88	9.88
**666.37689**	Positive	7.55	9.55	**726.40179**	Positive	7.77	9.77
**666.37909**	Positive	7.71	9.71	**730.39178**	Positive	7.63	9.63
**668.39374**	Positive	7.71	9.71	**730.39844**	Positive	7.52	9.52
**686.40308**	Positive	7.56	9.56	**732.37341**	Positive	5.82	7.82
**688.41754**	Positive	7.85	9.85	**732.37616**	Positive	5.41	7.41
**699.38922**	Positive	7.77	9.77	**732.40814**	Positive	7.03	9.03
**699.39215**	Positive	7.85	9.85	**734.38873**	Positive	4.83	6.83
**700.38251**	Positive	6.78	8.78	**734.38885**	Positive	5.90	7.90
**700.38361**	Positive	6.97	8.97	**734.38989**	Positive	5.36	7.36
**702.39832**	Positive	6.74	8.74	**734.39020**	Positive	5.28	7.28
**702.39948**	Positive	6.81	8.81	**734.39056**	Positive	5.09	7.09
**702.39954**	Positive	6.39	8.39	**734.39062**	Positive	5.79	7.79
**702.39960**	Positive	6.86	8.86	**734.42273**	Positive	7.85	9.85
**702.40222**	Positive	7.09	9.09	**736.40515**	Positive	7.82	9.82
**714.40143**	Positive	7.61	9.61	**736.40649**	Positive	7.57	9.57
**714.40308**	Positive	7.90	9.90	**744.40967**	Positive	7.58	9.58
**716.37708**	Positive	6.08	8.95	**746.4259**	Positive	7.77	9.77
**716.37781**	Positive	6.68	8.68	**748.36908**	Positive	5.59	7.59
**716.37866**	Positive	6.80	8.80	**762.41846**	Positive	7.57	9.57
**716.37982**	Positive	5.27	7.27	**762.4245**	Positive	7.68	9.68
**716.38098**	Positive	6.18	8.18	**720.41116**	Positive	7.00	9.00

Table S3. The mean relative abundance of lolitrem B biotransformation products distributed in the body and brain tissue of mice exposed to lolitrem B at high (2.0 mg/kg b.wt) and low (0.5 mg/kg b.wt) doses at time points 6 h and 24 h post treatment.

Compound	Lolitrem B[1]	L1	L2	L3	L4	L5	L6	L7	L8	L9	L10
**[M+H]+ m/z**	686.4036	702.4014	702.3971	618.3426	602.3470	602.3466	716.3779	716.3792	718.3946	718.3954	734.3923
**Retention time, min**	8.56	7.34	7.83	6.46	7.38	7.72	7.77	7.66	7.73	6.45	6.89
Kidney											
**High Dose 6 h**	10275508 *(n=7)*	325609 *(n=7)*	82340 *(n=6)*	6297 *(n=3)*	83863 *(n=7)*	8715 *(n=2)*	27560 *(n=4)*	114476 *(n=5)*	37896 *(n=7)*	91987 *(n=7)*	ND
**High Dose 24 h**	7923119 *(n=8)*	38878 *(n=8)*	19268 *(n=1)*	4552 *(n=1)*	10393 *(n=7)*	10220 *(n=1)*	7464 *(n=1)*	ND	ND	5706 *(n=3)*	ND
**Low Dose 6 h**	1714329 *(n=8)*	35471 *(n=7)*	15580 *(n=1)*	6152 *(n=3)*	9248 *(n=5)*	15148 *(n=1)*	9633 *(n=1)*	ND	ND	6336 *(n=3)*	ND
**Low Dose 24 h**	1342210 *(n=8)*	7044 *(n=3)*	ND	5732 *(n=2)*	6515 *(n=2)*	8067 *(n=1)*	9460 *(n=1)*	ND	8661 *(n=1)*	ND	ND
Liver											
**High Dose 6 h**	3510325 *(n=7)*	1381557 *(n=8)*	1647784 *(n=8)*	146661 *(n=8)*	447949 *(n=8)*	41949 *(n=6)*	451932 *(n=7)*	2182552 *(n=8)*	4184258 *(n=8)*	3254011 *(n=8)*	209101 *(n=7)*
**High Dose 24 h**	888700 *(n=8)*	141226 *(n=8)*	657443 *(n=8)*	20237 *(n=6)*	39645 *(n=8)*	ND	139136 *(n=8)*	90107 *(n=6)*	270427 *(n=8)*	783628 *(n=8)*	55205 *(n=8)*
**Low Dose 6 h**	517071 *(n=8)*	358957 *(n=8)*	381751 *(n=8)*	21101 *(n=6)*	113841 *(n=6)*	11835 *(n=2)*	45921 *(n=5)*	359662 *(n=6)*	1047318 *(n=8)*	927846 *(n=8)*	40885 *(n=6)*
**Low Dose 24 h**	180935 *(n=5)*	41020 *(n=4)*	100081 *(n=5)*	28173 *(n=2)*	9337 *(n=3)*	ND	14829 *(n=3)*	ND	20144 *(n=4)*	190591 *(n=5)*	11475 *(n=1)*
Cerebral cortex											
**High Dose 6 h**	35979 *(n=7)*	ND	ND	ND	ND	ND	ND	ND	ND	ND	ND
**High Dose 24 h**	40751 *(n=8)*	ND	ND	ND	ND	ND	ND	ND	ND	ND	ND
**Low Dose 6 h**	12822 *(n=3)*	ND	ND	ND	3858 *(n=1)*	ND	ND	ND	ND	ND	ND
**Low Dose 24 h**	14008 *(n=4)*	ND	ND	ND	ND	6418 *(n=1)*	ND	ND	ND	ND	ND

Thalamus											
**High Dose 6 h**	21943 *(n=6)*	5080 *(n=1)*	6085 *(n=1)*	ND	ND	6402 *(n=1)*	ND	ND	ND	ND	ND
**High Dose 24 h**	27861 *(n=6)*	ND	7151 *(n=1)*	ND	ND	ND	ND	6097 *(n=1)*	5350 *(n=2)*	ND	ND
**Low Dose 6 h**	18211 *(n=3)*	ND	5442 *(n=1)*	ND	ND	ND	ND	ND	5498 *(n=2)*	ND	ND
**Low Dose 24 h**	22645 *(n=7)*	ND	4729 *(n=1)*	ND	ND	ND	ND	5692 *(n=1)*	5708 *(n=2)*	ND	ND
Cerebellum											
**High Dose 6 h**	ND	6476 *(n=3)*	5515 *(n=1)*	ND	ND	ND	ND	7227 *(n=1)*	5526 *(n=3)*	ND	ND
**High Dose 24 h**	ND	6755 *(n=1)*	ND	ND	ND	ND	ND	ND	7325 *(n=1)*	6324 *(n=1)*	ND
**Low Dose 6 h**	ND	ND	6373 *(n=1)*	10010 *(n=1)*	ND	ND	5192 *(n=1)*	6859 *(n=2)*	ND	ND	ND
**Low Dose 24 h**	ND	ND	ND	ND	7931 *(n=1)*	ND	ND	5643 *(n=2)*	ND	8517 *(n=1)*	ND
Brainstem											
**High Dose 6 h**	ND	ND	5294 *(n=1)*	ND	ND	5793 *(n=1)*	ND	6617 *(n=2)*	ND	6334 *(n=4)*	ND
**High Dose 24 h**	ND	6197 *(n=1)*	ND	ND	ND	ND	ND	6299 *(n=1)*	ND	7016 *(n=3)*	ND
**Low Dose 6 h**	ND	ND	6015 *(n=2)*	ND	ND	ND	ND	7421 *(n=2)*	6201 *(n=1)*	ND	ND
**Low Dose 24 h**	ND	ND	6277 *(n=1)*	7966 *(n=2)*	ND	ND	ND	5946 *(n=1)*	ND	ND	ND

ND= Not detected, n = number of observations within the treatment group.

[1] Lolitrem B quantitative analysis is described in Reddy et al [13].
